# DHA status influences effects of B-vitamin supplementation on cognitive ageing: a post-hoc analysis of the B-proof trial

**DOI:** 10.1007/s00394-022-02924-w

**Published:** 2022-06-15

**Authors:** Annick P. M. van Soest, Ondine van de Rest, Renger F. Witkamp, Tommy Cederholm, Lisette C. P. G. M. de Groot

**Affiliations:** 1 Division of Human Nutrition and Health, Wageningen University & Research, P.O. Box 12, 6700 AA Wageningen, The Netherlands; 2 Department of Public Health and Caring Sciences, Clinical Nutrition and Metabolism, Uppsala University, Uppsala, Sweden

**Keywords:** B-vitamins, Omega-3 fatty acids, Cognition, Older adults, Elderly, Healthy ageing

## Abstract

**Purpose:**

Trials aiming to lower homocysteine by B-vitamin supplementation have reported mixed results on slowing cognitive decline. We investigated if efficacy of B-vitamin supplementation is affected by baseline plasma omega-3 fatty acid levels.

**Methods:**

This post-hoc analysis of the B-proof trial included 191 adults aged 65 years or older with baseline plasma total homocysteine ≥ 12 μmol/L, randomly assigned to 400 µg folic acid and 500 µg vitamin B12 or placebo daily for 2 years. Global and domain-specific cognitive functioning were assessed at baseline and after 2 years. The effect of B-vitamin supplementation was analyzed according to tertiles of baseline plasma omega-3 fatty acids concentrations combined, and eicosapentaenoic acid (EPA) and docosahexaenoic acid (DHA) individually using multiple linear regression analyses.

**Results:**

The mean ± SD age of the participants was 71.6 ± 5.9 years and median [IQR] Mini-Mental State Examination was 29 [28–30]. The treatment effect of B-vitamins on global cognition was larger in participants in the high compared to the middle DHA tertile (difference in z-score, mean ± SE 0.22 ± 0.10, *p* = 0.03). There was no significant interaction between B-vitamin supplementation and combined omega-3 fatty acid (*p* = 0.49) and EPA (*p* = 0.99) tertiles. Similarly, the efficacy of B-vitamin treatment on domain-specific cognitive functioning did not link to omega-3 fatty acid, DHA, or EPA plasma levels.

**Conclusion:**

This post-hoc analysis indicated that efficacy of B-vitamin supplementation in slowing cognitive decline relates to DHA status, with individuals with higher plasma DHA levels benefitting more from vitamin B12 and folic acid use. The results support earlier observations that positive effects of B-vitamins in cognitive ageing may be subgroup-specific.

**Trial registration**: Registered at clinicaltrials.gov (NCT00696514) on June 12, 2008.

**Supplementary Information:**

The online version contains supplementary material available at 10.1007/s00394-022-02924-w.

## Introduction

Age-related cognitive decline leading to dementia poses a societal challenge with major medical, social and economic impact. In the absence of curative treatment for dementia, the focus is on prevention by management of risk factors [1]. Epidemiological studies show that individuals with elevated homocysteine levels are at greater risk of cognitive decline and dementia, identifying homocysteine as a possible modifiable risk factor [2].

Elevated homocysteine levels may reflect impaired B-vitamin status [3]. B-vitamin supplementation to lower homocysteine levels and thereby slowing down cognitive decline would seem a straightforward solution, yet proof of clinical benefits is lacking. While clinical trials show that B-vitamin treatment, usually existing of vitamin B12, B6 and/or folic acid, is effective in lowering homocysteine levels, its effect on slowing down cognitive decline remains inconclusive [4].

It has been hypothesized that the efficacy of B-vitamin supplementation in slowing cognitive decline is dependent on omega-3 fatty acid status, with B-vitamin supplementation being only effective in individuals with higher omega-3 fatty acid plasma levels. Indeed, results from several post-hoc analyses of B-vitamin trials underline this hypothesis [5, 6]. Surprisingly, opposite results have been demonstrated as well, with only individuals with lower omega-3 fatty acid status benefitting from B-vitamin supplementation [7]. This merits further research to disentangle the complex interaction between B-vitamins and omega-3 fatty acids in cognitive ageing.

Thus, the current study further investigates the interaction between B-vitamin supplementation and omega-3 fatty acids with respect to cognitive outcomes in healthy older adults without cognitive complaints. To this end, we investigated if the efficacy of B-vitamin supplementation was dependent on baseline omega-3 fatty acid plasma levels in cognitively healthy older adults in the B-proof trial (B-Vitamins for the Prevention of Osteoporotic Fractures). In the main study of the B-proof trial, no effects of B-vitamins on slowing cognitive decline were observed [8].

## Methods

### Study design and participants

The present study was conducted as a post-hoc analysis within the B-proof trial, a randomized, double-blind placebo-controlled trial investigating the effect of folic acid and vitamin B12 supplementation on fracture incidence. Cognitive functioning was measured as secondary outcome. Data was collected between October 2008 and March 2013 in three research centers in the Netherlands: Erasmus Medical Center (Rotterdam), VU University Medical Center (Amsterdam) and Wageningen University (Wageningen). This analysis is based on a subsample of the Wageningen participants for whom fatty acid data were available. The trial has been approved by the Medical Ethics committee from Wageningen University & Research and has been registered at clinicaltrials.gov (NCT00696514). All participants provided written informed consent.

Information on study design and participants has been described in detail previously [9]. In short, the intervention existed of daily administration of 400 µg folic acid and 500 µg vitamin B12 tablets versus placebo tablets for a period of 2 years. Both intervention and placebo tablets contained 15 µg vitamin D_3_. Participants received tablets every 6 months and they were requested to return any remaining tablets, as a measure of compliance. Participants were men and women aged 65 years and older, with elevated plasma homocysteine levels (12–50 µmol/L). Exclusion criteria were renal insufficiency (creatinine > 150 µmol/L), diagnosis of a malignancy in the past 5 years and current or recent (< 4 months) use of supplements with very high dose of folic acid (> 300 µg) or intramuscular injections with vitamin B12. Fatty acid data was available for 205 participants. Our analysis included data of 191 participants. Data from 13 participants were excluded due to missing follow-up (*n* = 3) or ApoE4 (*n* = 10) data, and data from 1 participant was excluded due to a follow-up MMSE score of 19, indicating possible dementia.

### Cognitive testing

Cognitive functioning was assessed at baseline and after 2 years of intervention with an extensive battery of cognitive tests administered by trained research assistants.

In the *Rey Auditory Verbal Learning Test* (RAVLT) [10], a list of 15 words was verbally presented to the participant at a rate of one word per two seconds. The participant was asked to recall the words in five trials immediately after presentation (subtest immediate), and after a 20-min delay (subtest delayed). Subsequently, the participant was asked to identify the 15 words in a list of 30 verbally presented words (subtest recognition). The number of correctly recalled words in each subtest was recorded.

In the *Digit Span Task* [11], the participant was verbally presented with digit sequences and asked to recall the sequence in either forward or backward order. Starting at a sequence length of three digits in the forward and two digits in the backward task, the length increased each two trials until an error was made or the maximum length of nine digits in the forward and eight in the backward task was reached. The maximum sequence length for the forward and backward version was recorded.

In the *Trail Making Test* (TMT) [12], participants were presented with a paper containing 25 circles. In two subtests, participants were asked to connect 25 circles containing numbers in chronological order (part A), and to alternate connecting circles containing numbers and letters in chronological and alphabetical order (part B). Time to complete each part was recorded.

The *Stroop Colour-Word test* [13] exists of three subtests, in which the participant was presented with colour words written in black ink (part I), coloured blocks (part II), or colour words written in an incongruent colour ink (part III). The participant is instructed to read aloud the words as fast as possible. The time needed to complete each part was documented.

In the *Symbol Digit Modalities Test (SDMT) *[14], symbols were paired with digits. The participant was presented with a sheet of symbols, and asked to match the symbols to the corresponding digit as fast as possible. The number of correctly matched pairs in 90 s was recorded.

In *Letter Fluency *[15], participants were given 60 s to name as many words as possible starting with the letter D, A and T (baseline) or K, O, and M (follow-up). The number of unique words was documented.

Parallel versions were used for RAVLT, TMT and Verbal Fluency to minimize learning effects. Individual cognitive test scores at baseline and follow-up were converted into Z-scores based on baseline mean and standard deviation, with higher scores indicating better cognitive functioning. The Z-scores for TMT and Stroop Colour-Word test were reversed as lower scores indicate better cognitive functioning. Individual Z-scores were clustered into composite scores for global and domain specific cognitive functioning:

$$\begin{aligned} \mathrm{Global \; cognition } & = ({Z}_{\mathrm{RAVLTimmediate}}+{Z}_{\text{RAVLTdelayed}}+{Z}_{\text{RAVLTrecognition}}+{Z}_{\mathrm{DigitSpan \; forward}}+{Z}_{\mathrm{DigitSpan \; backward}} \\& \quad + -{Z}_{\mathrm{Stroop \; mean \; I \; and \;II}}+ {-Z}_{\text{TMTpartA}}+{Z}_{\text{SDMT}}+ {-Z}_{\mathrm{Stroop \; interference}}+{-Z}_{\text{TMTB/A}} +{Z}_{\text{Fluency}})/ 11\end{aligned}$$

$$\text{Episodic} \; \text{memory}=\left({Z}_{\text{RAVLTimmediate}}+{Z}_{\text{RAVLTdelayed}}+{Z}_{\text{RAVLTrecognition}}\right)/3$$

$$\mathrm{Attention } \; \& \; \mathrm{working \; memory}=({Z}_{\mathrm{DigitSpan \; forward}}+{Z}_{\mathrm{DigitSpan \; backward}})/2$$

$$\mathrm{Information \; processing \; speed}=(-{Z}_{\mathrm{Stroop \; mean \; I \;and \;II}}+ {-Z}_{\text{TMTpartA}}+{Z}_{\text{SDMT}})/ 3$$

$$\mathrm{Executive \; functioning }=({-Z}_{\text{Stroop} \; \text{interference}}+{-Z}_{\text{TMTB/A}} +{Z}_{\text{Fluency}})/ 3$$

### Biochemical assays

Baseline omega-3 fatty acid concentrations were measured in the plasma phospholipid (PL) fractions from blood samples obtained after an overnight fast or a light breakfast. Samples had been collected by venipuncture using EDTA containing vacuum tubes. Plasma was obtained by centrifugation (10 min at 1200*g*) and stored at − 80 °C. Studies have shown that polyunsaturated fatty acids remain stable for up to 12 years under these conditions [16]. Total lipids were extracted from plasma with isopropanol/hexane (2:3, v/v) and separated into cholesteryl and PL fractions by solid phase extraction using silica columns. Subsequently, fatty acids in the PL fractions were transesterified using boron trifluoride in methanol yielding their methyl esters. Analysis was performed by gas chromatography with flame-ionization detection. Peaks were identified based on comparison of retention times to known standards. Fatty acid concentrations are presented in relative concentrations of total fatty acids. The relative concentration of plasma omega-3 fatty acids was derived by adding the proportions of eicosapentaenoic acid (EPA) and docosahexaenoic acid (DHA). A detailed description of the analytical procedure for fatty acids used in our lab has been published elsewhere [17].

Serum vitamin B12 and folate were determined using immune electrochemiluminescence assay (Elecsys, 2010, Roche). High-performance liquid chromatography was used to measure plasma total homocysteine [18]. DNA was isolated from buffy coats for genotyping. ApoE genotype was determined using TaqMan analysis.

### Descriptive characteristics

Trained research assistants measured height with a stadiometer to the nearest 0.1 cm and weight with a calibrated scale to the nearest 0.5 kg. Body mass index (BMI) was calculated as weight (kg)/(height (m))^2^. Information on age, sex, education level (low, middle, high), smoking status (never, former, current) and physical activity [19] was obtained via questionnaires. MMSE (0–30 points) [20] was assessed by trained research assistants following a standardized protocol.

### Statistical analysis

Data are expressed as *n* (%), mean (SD) or median (IQR) unless otherwise stated.

Baseline characteristics between intervention and omega-3 fatty acid groups were compared using independent sample *t*-test, ANOVA or Kruskal–Wallis test for continuous variables and chi-square for categorical variables. Multiple linear regression was performed to investigate if the efficacy of B-vitamin supplementation was dependent on baseline omega-3 fatty acid levels. We modelled the change in cognition Z-score between baseline and post-intervention for global cognition and domain-specific cognition as a function of intervention group (B-vitamins, placebo), baseline omega-3 fatty acid group (low, middle and high) and their interaction. To investigate if DHA and/or EPA status modified B-vitamin supplementation efficacy separately, additional models were run replacing baseline omega-3 fatty acid levels (by groups) by either DHA or EPA concentrations (by groups). To create the omega-3 fatty acid status groups, baseline omega-3 fatty acid, DHA and EPA concentrations were divided into tertiles. The analyses were adjusted for baseline cognitive Z-score, age, sex, education, ApoE4 status, baseline homocysteine level, physical activity and smoking status, all measured at baseline. Tukey correction for multiple comparisons was applied when examining treatment effects within the omega-3 fatty acid tertiles. *p*-values < 0.05 were considered statistically significant, for interaction terms the cut-off was set at *p* < 0.10. All analyses were performed using RStudio Version 1.1.463 [21].

## Results

### Participant characteristics

Table 1 presents baseline characteristics of the study population. The mean age of the participants was 71.5 ± 5.8 years and 56% was male. The average BMI was 27.5 ± 4.2 kg/m^2^, with 76% being overweight (i.e. BMI ≥ 25 kg/m^2^). Total homocysteine levels were elevated with a median of 13.7 [IQR 12.9–15.8] µmol/L. The study population was cognitively healthy, as indicated by a median MMSE score of 29 [IQR 28–30] at baseline. Five participants (2.6%) had MMSE scores equal to or lower than 24, indicating cognitive impairment. Participants in the B-vitamin group were younger (*p* = 0.01) compared to participants in the placebo group. Furthermore, a larger proportion of participants in the middle omega-3 fatty acid tertile had never smoked compared to participants in the high omega-3 fatty acid tertile (*p* = 0.02) (Supplementary Table 1). Mean baseline cognitive scores did not differ between either intervention or omega-3 fatty acid status groups. Compliance to treatment was high with an average of 97%. There was no difference in compliance between treatment and/or omega-3 fatty acid groups.

**Table 1:** Baseline characteristics per treatment group in the B-proof study

Characteristic	Overall (*n* = 191)	B vitamin (*n* = 94)	Placebo (*n* = 97)	*p*-value
Age (years)	71.5 ± 5.8	70.3 ± 5.1	72.7 ± 6.3	> 0.01
Sex *n* (%)				
Male	107 (56%)	52 (55%)	55 (57%)	0.96
Female	84 (44%)	42 (45%)	42 (43%)	
Level of education *n* (%)				0.27
Low	76 (40%)	41 (43%)	35 (36%)	
Middle	46 (24%)	18 (19%)	28 (29%)	
High	69 (36%)	35 (37%)	34 (35%)	
BMI (kg/m^2^)	27.5 ± 4.2	27.5 ± 4.4	27.5 ± 4.0	0.95
Physical activity (kcal/d)	561 (358–863)	596 (386–879)	525 (326–810)	0.09
Smoking behaviour *n* (%)				0.54
Current smoker	11 (6%)	7 (7%)	4 (4%)	
Former smoker	123 (64%)	61 (65%)	62 (64%)	
Never smoker	57 (30%)	26 (28%)	31 (32%)	
ApoE4 carriers *n* (%)	55 (29%)	28 (30%)	27 (28%)	0.89
Biochemical measures				
Total homocysteine (µmol/L)	13.7 (12.9–15.8)	13.7 (13.0–15.3)	13.7 (12.9–16.4)	0.65
Folate (nmol/L)	17.4 (14.1–23.5)	16.9 (13.9–22.4)	17.7 (14.3–24.7)	0.12
Vitamin B12 (pmol/L)	256 (201–334)	253 (203–308)	275 (196–366)	0.10
MMA (μmol/L)	0.22 (0.19–0.29)	0.22 (0.19–0.29)	0.22 (0.19–0.31)	0.80
holoTC (pmol/L)	62 (46–80)	63 (47–76)	62 (46–82)	0.73
25(OH)D (nmol/L)	60 ± 23	61 ± 24	60 ± 22	0.84
Omega-3 status (sum DHA and EPA, %)^a^	5.7 ± 1.9	5.5 ± 1.8	5.9 ± 2.1	0.20
DHA (%)^a^	4.3 ± 1.2	4.2 ± 1.2	4.5 ± 1.3	0.11
EPA (%)^a^	1.4 ± 0.9	1.3 ± 0.8	1.4 ± 1.0	0.54
MMSE score	29 (28–30)	29 (27–30)	29 (28–30)	0.85
Global cognition *Z*-score	0.00 ± 0.52	0.01 ± 0.54	− 0.02 ± 0.51	0.69
Episodic memory *Z*-score	0.00 ± 0.70	0.03 ± 0.70	− 0.02 ± 0.70	0.59
Attention and working memory *Z*-score	0.00 ± 0.86	0.01 ± 0.89	− 0.00 ± 0.85	0.94
Information processing speed *Z*-score	0.00 ± 0.77	− 0.02 ± 0.79	0.02 ± 0.76	0.73
Executive functioning *Z*-score	0.00 ± 0.69	0.04 ± 0.69	− 0.05 ± 0.69	0.37

B-proof subjects with available fatty acid and cognition data at both time points

BMI: body mass index, MMA: methylmalonic acid, holoTC: holotranscobalamin, DHA: docosahexaenoic acid, EPA: eicosapentaenoic acid, MMSE: Mini Mental State Examination. Data are mean ± SD, median (IQR) or number (%)

^a^Measured in phospholipid fractions

Comparing our subsample with the total Wageningen and B-proof study populations, our subsample was younger than the Wageningen (72.9 ± 5.7, *p* < 0.01) and B-proof (74.3 ± 6.6y, *p* < 0.01) study populations. Median Mini-Mental State Examination (MMSE) score was similar in our subsample and the total Wageningen study population (29 [28–30] for both, *p* = 0.46). The total B-proof study population showed lower MMSE scores (28 [27–29], *p* < 0.01).

### Cognitive performance

#### Global cognitive functioning

**Fig. 1:**
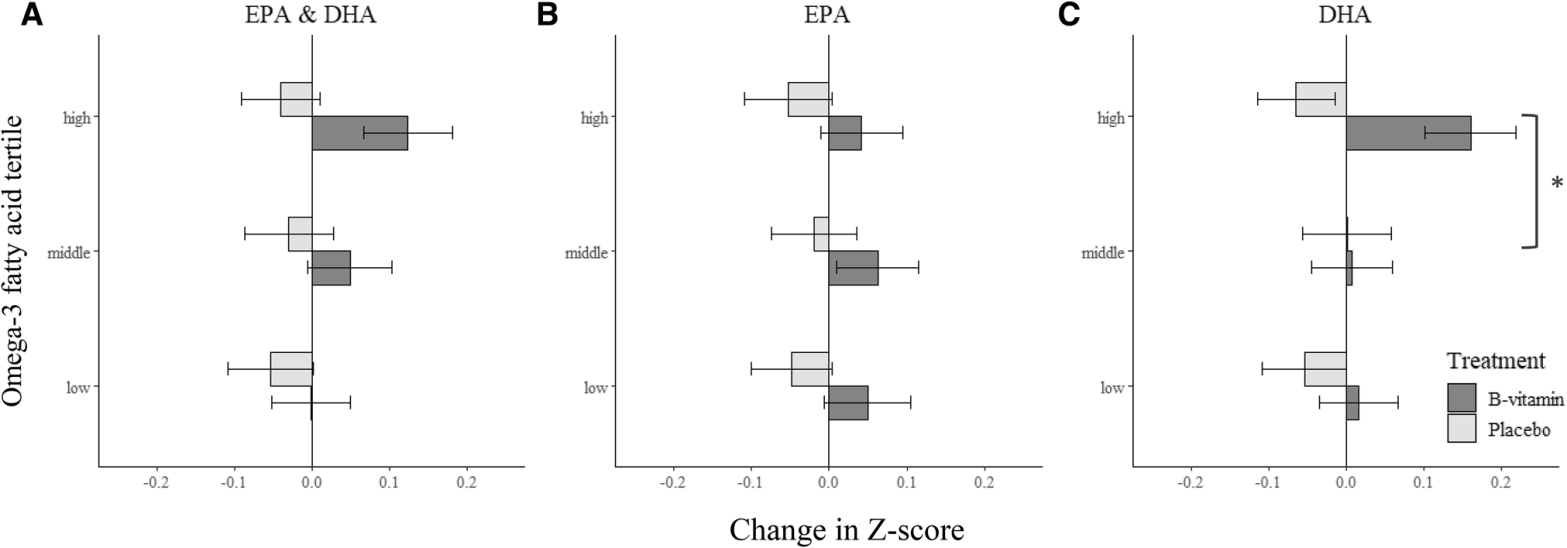
Changes in Z-scores (mean *β* ± SE) in global cognition over the two year intervention period according to treatment group and **A** omega-3 fatty acid status, **B** eicosapentaenoic acid (EPA) status and **C** docosahexaenoic acid (DHA) status. *Significant (*p* < 0.05) difference in treatment effect between omega-3 fatty acid tertiles, as analyzed by linear multiple regression

The treatment effects of B-vitamins versus placebo on global cognition were numerically positive (i.e. larger than 0) in all omega-3 fatty acid (EPA and DHA combined) tertiles, indicating that the group that received B-vitamins improved more over time compared to the placebo group, irrespective of the omega-3 fatty acid blood levels (Table 2; Fig. 1). Despite the larger treatment effect in participants in the high omega-3 fatty acid tertile (difference 0.16 ± 0.07, *p* = 0.25) compared to the middle and low tertiles (respectively, 0.08 ± 0.07, *p* = 0.75; 0.05 ± 0.07, *p* = 0.97), there was no significant overall interaction between B-vitamin supplementation and omega-3 fatty acid tertile (*p* = 0.60), meaning that there is no difference in treatment effect of B-vitamins between the low, middle and high omega-3 fatty acid tertiles.

**Table 2:** Changes in global cognition Z-scores following B-vitamin versus placebo supplementation according to omega-3 fatty acid, EPA and DHA status tertile

	Treatment effect^a^			Overall interaction^b^	Tertiles pairwise comparison^c^		
	Crude	Adjusted	*p*-value	*p*-value	Low vs middle	Low vs high	Middle vs high
Omega-3 fatty acid status				0.60			
Low tertile	0.10 ± 0.07	0.05 ± 0.07	0.97		diff = 0.04 ± 0.10 *p* = 0.69	diff = 0.10 ± 0.10 *p* = 0.32	diff = 0.06 ± 0.10 *p* = 0.54
Middle tertile	0.13 ± 0.07	0.08 ± 0.07	0.75				
High tertile	0.16 ± 0.07	0.16 ± 0.07	0.25				
EPA status				0.97			
Low tertile	0.16 ± 0.07	0.10 ± 0.07	0.72		diff = 0.02 ± 0.10 *p* = 0.82	diff = 0.02 ± 0.10 *p* = 0.98	diff = 0.02 ± 0.10 *p* = 0.84
Middle tertile	0.09 ± 0.07	0.07 ± 0.07	0.88				
High tertile	0.12 ± 0.07	0.09 ± 0.07	0.75				
DHA status				0.06			
Low tertile	0.12 ± 0.07	0.06 ± 0.07	0.95		diff = 0.05 ± 0.10 *p* = 0.59	diff = 0.18 ± 0.10 *p* = 0.07	diff = 0.23 ± 0.10 *p* = 0.02
Middle tertile	0.04 ± 0.07	0.01 ± 0.07	1.00				
High tertile	0.24 ± 0.07	0.24 ± 0.07	0.01				

Data available for n = 191 participants. Data is presented as mean *β* ± SEM

EPA: eicosapentaenoic acid; DHA: docosahexaenoic acid

^a^Treatment effect is the difference in change in Z-score over time between the B-vitamin and placebo treatment groups within an omega-3 fatty acid tertile as analyzed using linear multiple regression, equal to Δ Z-score B-vitamin − Δ Z-score placebo

Crude model: adjusted for baseline cognitive Z-score; Adjusted model: adjusted for baseline cognitive Z-score, age, sex, level of education, ApoE4 status, baseline homocysteine concentration, baseline body mass index, physical activity, smoking status

^b^The overall interaction indicates similarity of treatment effects in the low, middle and high omega-3 fatty acid tertiles

^c^The pairwise comparison tests for differences in treatment effects between omega-3 fatty acid tertiles

Subsequently, we analysed the treatment effects related to EPA and DHA concentrations separately. The efficacy of B-vitamin supplementation related to plasma DHA levels. B-vitamin supplementation was more effective than placebo in maintaining global cognitive functioning in participants in the high DHA tertile (difference 0.24 ± 0.07, *p* = 0.01), while no difference in treatment effect was observed in participants in the middle or low DHA tertile (respectively, *p* = 1.00 and *p* = 0.95). The overall interaction between B-vitamin supplementation and DHA status was significant (*p* = 0.06). Participants in the high DHA tertile benefited significantly more from B-vitamin supplementation compared to participants in the middle DHA tertile (difference 0.23 ± 0.10, *p* = 0.02). Furthermore, there was a trend towards a difference in treatment effect between the high and low DHA tertile (0.18 ± 0.10 *p* = 0.07).

Corresponding analyses for potential interaction with EPA, i.e. comparing B-vitamin and placebo supplementation for EPA status, revealed that no treatment effect was observed in any of the EPA groups. In addition, there was no significant overall interaction between B-vitamin supplementation and EPA status (*p* = 0.97).

#### Domain-specific cognitive functioning

For none of the four cognitive domains separately; i.e. episodic memory, attention & working memory, information processing speed, and executive functioning, there was a difference in treatment effect in any of the combined omega-3 fatty acid groups (Supplementary Table 2). In addition, there was no significant overall interaction between B-vitamin supplementation and omega-3 fatty acid group. Similarly, when domain-specific performance was assessed in relation to tertiles of baseline concentrations of EPA and DHA individually, there were no significant treatment effects or interactions (Supplementary Tables 3, 4).

## Discussion

This post-hoc analysis of the B-proof trial showed that the efficacy of B-vitamin supplementation on global cognition may be related to plasma DHA levels, but not to plasma total omega-3 fatty acid or EPA levels. Individuals with higher DHA plasma levels benefitted from B-vitamin supplementation, while individuals with lower DHA plasma levels did not. With respect to domain-specific cognitive performance, plasma omega-3 fatty acid combined, DHA or EPA levels separately did not modify the treatment effect of B-vitamins on episodic memory, attention & working memory, information processing speed nor executive functioning.

To date, the interaction between B-vitamins and omega-3 fatty acids in relation to cognitive decline have been investigated in three post-hoc analyses and one clinical trial, with mostly similar [5, 6, 22, 23] but also contrasting [7] findings. In line with our results, the VITACOG trial, in which older adults (> 70y) with MCI were supplemented with B-vitamins (folic acid, vitamin B6 and B12) versus placebo for 2 years, showed that omega-3 fatty acid status influenced B-vitamin treatment efficacy. Only individuals with higher plasma omega-3 fatty acid levels showed slower rates of cognitive decline [5] and brain atrophy [6] following B-vitamin supplementation. Similarly, a post-hoc analysis of the OmegAD randomized controlled trial showed that adequate levels of both omega-3 fatty acids and B-vitamins are needed [22]. In the OmegAD trial on the effect of 6 month daily supplementation with EPA and DHA versus placebo in AD patients, only subjects with lower homocysteine status benefited from omega-3 fatty acid supplementation. Further proof comes from a recent randomized controlled trial with a factorial design, in which older adults with MCI were supplemented with placebo, 0.8 mg folic acid, 0.8 mg DHA or a combination of the two daily for 6 months. Combined intervention of folic acid and DHA was more effective in improving cognition compared to supplementation with only folic acid or DHA, adding proof for the interaction from a factorial clinical trial [23].

Contrary to our current results and previous studies, the post-hoc analysis of the FACIT trial [7], performed by our group, showed that either sufficient availability of omega-3 fatty acids or B-vitamins may be needed. In this randomized controlled trial on the effect of 3-year daily supplementation with folic acid in cognitively healthy middle-aged adults (50–70 years) with elevated plasma homocysteine, folic acid supplementation was only beneficial in improving cognition in individuals with lower omega-3 fatty acid status, while individuals with higher omega-3 fatty acid status did not experience benefits.

The B-proof, VITACOG and OmegAD trials differed from the FACIT trial on various different aspects that could potentially explain the opposite findings. Importantly, B-proof, VITACOG and OmegAD participants were older, with an average age of over 70, versus an average age of 60 in the FACIT trial. In older individuals, needs for omega-3 fatty acids may be higher due to changes in dietary intake, bioavailability and increased membrane synthesis rates, as discussed previously [7]. Additionally, baseline omega-3 fatty acid status could be different between study populations, yet no direct comparison can be made due to differences in the fatty acid fractions analyzed, analytical methods and expressed measures. However, the omega-3 fatty acid distribution of our study population is similar to that of other study populations from European (non-Scandinavian) countries [24]. Vitamin B12 status also differed between study populations, as in the FACIT trial individuals with vitamin B12 deficiency were excluded. In our previous publication, we hypothesized that the contrasting findings of the FACIT trial could be attributed to differences in baseline homocysteine status and/or type of B-vitamin intervention. These factors now seem less probable, as homocysteine levels were both elevated in FACIT and B-proof trials and B-vitamin treatment included only folic acid in both the FACIT trial and in the clinical trial of Li and colleagues [7, 23]. We strongly encourage researchers with access to data on both B-vitamin and omega-3 fatty acid status to perform post-hoc analyses to be able to better define populations that may benefit from a combination of B-vitamins and omega-3 fatty acids. These results can be the basis for the design of future clinical trials with a factorial design (comparing B-vitamin supplementation only, omega-3 fatty acid supplementation only, combined supplementation versus placebo).

A mechanistic explanation for the finding that B-vitamin supplementation was more effective in individuals with higher DHA status, may involve the interaction of B-vitamins with phospholipid metabolism [25]. Phosphatidylcholine (PC) plays a crucial role in the transport of omega-3 fatty acids, including DHA, to the brain. Interestingly, B-vitamins can influence the formation of PC [25]. In the one-carbon metabolism, the B-vitamins folic acid, B6 and B12 play an important role in regulating homocysteine levels. Inadequate B-vitamin status results in elevated levels of homocysteine and its precursor, S-adenosyl homocysteine (SAH) [26]. In turn, the accumulation of SAH slows down the enzyme phosphatidylethanolamine-N-methyltransferase, which converts phosphatidylethanolamine to PC [25]. In short, adequate B-vitamin status is needed to ensure sufficient PC production, and thus transport of omega-3 fatty acids to the brain. To support this possible mechanistic explanation, for further research it would be interesting to measure the proportion of omega-3 fatty acids bound to PC.

Here we demonstrated that DHA status, but not EPA or total omega-3 fatty acid status, modified efficacy of B-vitamin supplementation. An explanation may again involve the regulatory role of B-vitamins for omega-3 fatty acid transport to the brain. EPA and DHA have different mechanisms to promote brain health. While EPA is particularly known for its anti-inflammatory effects and is only present in the brain in limited amounts, DHA is the most abundant fatty acid in the brain. This omega-3 fatty acid increases membrane fluidity which is critical for synaptic vesicles and transmission of signals, demonstrating the importance of adequate DHA levels in the brain for proper functioning of the neuronal membrane [27]. Alternatively, the differences in study populations (cognitively healthy versus MCI) and treatment (dose, combination of B-vitamins versus folic acid) between our study and previous studies, may be responsible for the lack of interaction with EPA in the current study.

The current analyses were limited to the interaction between vitamin B12/folic acid and omega-3 fatty acids, yet there are indications that also other nutrients may be involved. Bowman and colleagues [28] demonstrated a possible role for vitamin D, by showing that adequate vitamin D status further enhances the protective effect of sufficient homocysteine and omega-3 fatty acid levels in cognitive ageing. Additionally, omega-3 fatty acids may interact with antioxidants: a post-hoc analysis of an antioxidant supplementation trial demonstrated that the association of omega-3 fatty acid intake with cognitive functioning was modulated by a multi-nutrient antioxidant supplement containing ascorbic acid, vitamin E, beta-carotene, selenium and zinc [29], illustrating the importance of a multi-nutrient approach in slowing down cognitive ageing. For the current study, although we did have dietary and blood nutrient assessment data available, unfortunately we were limited by our sample size to further look into the role of other nutrients in the interaction. Further research with larger sample size should consider incorporating vitamin D status and/or antioxidant intake and status.

A major limitation of the current post-hoc analysis is that we performed exploratory analyses not designed and adequately powered to investigate the modifying potential of omega-3 fatty acid status on B-vitamin supplementation efficacy. The small sample size may be responsible for the lack of findings for domain-specific cognitive functioning, and for the lack of significant differences between the low and high DHA tertiles. Additionally, omega-3 fatty acid status was only determined at baseline and in plasma phospholipids rather than red blood cells, which is a better proxy for long-term omega-3 fatty acid status. However, we assume that our measurements do represent longer-term status as dietary patterns (and thus omega-3 intake) in older adults are reasonably stable over time [30], and other factors that may influence variation (e.g. geographic and genetic reasons) also have remained stable. Though the 2-year duration of the trial is a fairly short period of time to recognize cognitive deteriorations in healthy older individuals, it can still be considered a strength as an intervention period of 2 years is quite long in comparison with other nutrition intervention studies to slow cognitive decline. Another strength of the study is the use of an extensive cognitive test battery with a focus on domain-specific tests, instead of general tests such as the MMSE or Telephone Interview for Cognitive Status.

In conclusion, this post-hoc analysis demonstrated that B-vitamin supplementation effectiveness in cognitive ageing is related to plasma DHA levels, with older adults with higher plasma DHA levels benefitting more from B-vitamin supplementation. The results support earlier observations that positive effects of B-vitamins in cognitive ageing may be subgroup-specific. Further research is needed to optimize defining subgroups that may be susceptible for B-vitamin supplementation, and subsequently to confirm this finding in a clinical trial with a factorial design.

## Supplementary Information

Below is the link to the electronic supplementary material.

Supplementary file 1 (DOCX 37 KB)

## Data Availability

Data and code are available upon reasonable request in consultation with the study team.

## Abbreviations

AD: Alzheimer’s disease
BMI: Body mass index
B-proof: B-vitamins for the prevention of osteoporotic fractures
DHA: Docosahexaenoic acid
EPA: Eicosapentaenoic acid
MCI: Mild cognitive impairment
MMSE: Mini mental state examination
PC: Phosphatidylcholine
RAVLT: Rey auditory verbal learning test
SDMT: Symbol digit modalities test
TMT: Trail making test
