# Supplementary material for: DHA status influences effects of B-vitamin supplementation on cognitive ageing: a post-hoc analysis of the B-proof trial
Source: Eur J Nutr. 2022 Jun 15;61(7):3731–9. doi: 10.1007/s00394-022-02924-w (PMC9464144; doi:10.1007/s00394-022-02924-w)
Supplement: Supplementary file 1 — (DOCX 37 KB) [file 394_2022_2924_MOESM1_ESM.docx]

**Supplementary information**

**DHA status influences effects of B-vitamin supplementation on cognitive ageing: a post-hoc analysis of the B-proof trial**

A.P.M. van Soest^1^ , O. van de Rest^1^, R.F. Witkamp^1^, T. Cederholm^2^ , L.C.P.G.M. de Groot^1^

^1^ Division of Human Nutrition and Health, Wageningen University & Research, Wageningen, The Netherlands

^2^ [Department of Public Health and Caring Sciences, Clinical Nutrition and Metabolism, Uppsala University](https://www.scopus.com/affil/profile.uri?afid=60003858), Uppsala, Sweden

Corresponding author: Annick van Soest, P.O. Box 12 6700 AA Wageningen The Netherlands, +31 317 488 077, annick.vansoest@wur.nl

**Supplementary table 1**: Baseline characteristics per omega-3 fatty acid tertile in the B-proof study^1^

| Characteristic | | Overall (n=191) | Low (n=64) | Middle (n=64) | High (n=63) | p-value |
| --- | --- | --- | --- | --- | --- | --- |
| Age (years) | | 71.5±5.8 | 72.1±6.6 | 70.7±4.8 | 71.8±6.0 | 0.36 |
| Sex n (%) | |  |  |  |  | 0.67 |
|  | Male | 107 (56%) | 33 (52%) | 37 (58%) | 37 (59%) |  |
|  | Female | 84 (44%) | 31 (48%) | 27 (42%) | 26 (41%) |  |
| Level of education n (%) | |  |  |  |  | 0.84 |
|  | Low | 76 (40%) | 28 (44%) | 26 (41%) | 22 (35%) |  |
|  | Middle | 46 (24%) | 12 (19%) | 17 (27%) | 17 (27%) |  |
|  | High | 69 (36%) | 24 (38%) | 21 (33%) | 24 (38%) |  |
| BMI (kg/m^2^) | | 27.5±4.2 | 26.6±4.0 | 28.1±4.5 | 27.9±3.9 | 0.08 |
| Physical activity (kcal/d) | | 561 (358-863) | 565 (356-943) | 584 (361-864) | 551 (374-815) | 0.82 |
| Smoking behavior n (%) | |  |  |  |  |  |
|  | Current smoker | 11 (6%) | 7 (11%) | 1 (2%) | 3 (5%) | 0.02 |
|  | Former smoker | 123 (64%) | 39 (61%) | 37 (58%) | 47 (75%) |  |
|  | Never smoker | 57 (30%) | 18 (28%) | 26 (41%) | 13 (21%) |  |
| ApoE4 carriers n (%) | | 55 (29%) | 18 (28%) | 13 (20%) | 24 (38%) | 0.09 |
| Biochemical measures | |  |  |  |  |  |
| Total homocysteine (µmol/L) | | 13.7 (12.9-15.8) | 14.1 (13.3-15.9) | 13.6 (12.8-16.0) | 13.6 (12.9-15.3) | 0.22 |
| Folate (nmol/L) | | 17.4 (14.1-23.5) | 16.7 (13.4-22.7) | 18.4 (14.9-24.4) | 17.6 (14.1-24.0) | 0.32 |
| Vitamin B12 (pmol/L) | | 256 (201-334) | 254 (200-304) | 257 (197-329) | 280 (224-369) | 0.18 |
| MMA (μmol/L) | | 0.22 (0.19-0.29) | 0.23 (0.19-0.32) | 0.22 (0.18-0.27) | 0.22 (0.19-0.28) | 0.36 |
| holoTC (pmol/L) | | 62 (46-80) | 58 (46-71) | 61 (46-77) | 66 (48-96) | 0.14 |
| 25(OH)D (nmol/L) | | 60±23 | 60±21 | 63±26 | 58±23 | 0.48 |
| Omega-3 status  (sum DHA and EPA, %)* | | 5.7±1.9 | 4.0±0.7 | 5.3±0.4 | 7.8±1.9 | <0.001 |
| DHA (%)* | | 4.3±1.2 | 3.1±0.6 | 4.2±0.4 | 5.6±0.9 | <0.001 |
| EPA (%)* | | 1.4±0.9 | 0.9±0.3 | 1.1±0.3 | 2.2±1.2 | <0.001 |
| MMSE score | | 29 (28-30) | 29 (27-29) | 29 (28-30) | 29 (27-30) | 0.83 |
| Global cognition Z-score | | 0.00±0.52 | 0.02±0.54 | 0.00±0.54 | -0.03±0.50 | 0.90 |
| Episodic memory Z-score | | 0.00±0.70 | 0.07±0.72 | 0.08±0.75 | -0.13±0.62 | 0.19 |
| Attention & working memory Z-score | | 0.00±0.86 | -0.09±0.88 | 0.00±0.80 | 0.09±0.91 | 0.52 |
| Information processing speed Z-score | | 0.00±0.77 | -0.00±0.82 | 0.03±0.76 | -0.03±0.75 | 0.89 |
| Executive functioning Z-score | | 0.00±0.69 | 0.06±0.64 | -0.09±0.71 | 0.03±0.71 | 0.41 |

^1^ B-proof subjects with available fatty acid and cognition data at both time points. Abbreviations: BMI: body mass index, MMA: methylmalonic acid, holoTC: holotranscobalamin, DHA: docosahexaenoic acid, EPA: eicosapentaenoic acid, MMSE: Mini Mental State Examination. Data are mean±SD, median (IQR) or number (%).

* measured in phospholipid fractions

**Supplementary table 2:** Changes in domain-specific cognitive Z-scores following B-vitamin versus placebo supplementation according to omega-3 fatty acid status tertile.

|  |  | Treatment effect^1^ | | | Overall interaction^2^ | Tertiles pairwise comparison^3^ | | |
| --- | --- | --- | --- | --- | --- | --- | --- | --- |
|  |  | Crude | Adjusted | p-value | p-value | low vs middle | low vs high | middle vs high |
| Episodic memory | |  |  |  | 0.99 |  |  |  |
|  | Low tertile | 0.17±0.15 | 0.10±0.15 | 0.98 |  | diff=0.03±0.21 p=0.89 | diff=0.00±0.21 p=0.99 | diff=0.03±0.21 p=0.90 |
|  | Middle tertile | 0.19±0.14 | 0.13±0.15 | 0.95 |  |  |  |  |
|  | High tertile | 0.07±0.15 | 0.11±0.15 | 0.98 |  |  |  |  |
| Attention & working memory | |  |  |  | 0.78 |  |  |  |
|  | Low tertile | 0.00±0.15 | 0.03±0.16 | 1.00 |  | diff=0.10±0.23 p=0.65 | diff=0.05±0.23 p=0.82 | diff=0.15±0.22 p=0.49 |
|  | Middle tertile | -0.06±0.15 | -0.07±0.16 | 0.99 |  |  |  |  |
|  | High tertile | 0.08±0.15 | 0.08±0.16 | 1.00 |  |  |  |  |
| Information processing speed | |  |  |  | 0.61 |  |  |  |
|  | Low tertile | 0.04±0.12 | 0.02±0.12 | 1.00 |  | diff=0.15±0.17 p=0.38 | diff=0.01±0.18 p=0.94 | diff=0.14±0.17 p=0.41 |
|  | Middle tertile | 0.28±0.12 | 0.17±0.12 | 0.73 |  |  |  |  |
|  | High tertile | 0.11±0.13 | 0.03±0.12 | 1.00 |  |  |  |  |
| Executive functioning | |  |  |  | 0.38 |  |  |  |
|  | Low tertile | 0.17±0.13 | 0.10±0.13 | 0.97 |  | diff=0.08±0.18 p=0.65 | diff=0.16±0.18 p=0.38 | diff=0.25±0.18 p=0.17 |
|  | Middle tertile | 0.06±0.13 | 0.02±0.13 | 1.00 |  |  |  |  |
|  | High tertile | 0.29±0.13 | 0.26±0.13 | 0.34 |  |  |  |  |

Data available for n=191 participants. Data is presented as mean β±SEM.

^1^ Treatment effect is the difference in change in Z-score over time between the B-vitamin and placebo treatment groups within an omega-3 fatty acid tertile as analyzed using linear multiple regression, equal to Δ Z-score B-vitamin - Δ Z-score placebo.

Crude model : adjusted for baseline cognitive Z-score; Adjusted model: adjusted for baseline cognitive Z-score, age, sex, level of education, ApoE4 status, baseline homocysteine concentration, baseline body mass index, physical activity, smoking status.

^2^ The overall interaction indicates similarity of treatment effects in the low, middle and high omega-3 fatty acid tertiles.

^3^ The pairwise comparison tests for differences in treatment effects between omega-3 fatty acid tertiles.

**Supplementary table 3:** Changes in domain-specific cognitive Z-scores following B-vitamin versus placebo supplementation according to EPA status tertile.

Data available for n=191 participants. Data is presented as mean β±SEM. Abbreviations: EPA: eicosapentaenoic acid

^1^ Treatment effect is the difference in change in Z-score over time between the B-vitamin and placebo treatment groups within an omega-3 fatty acid tertile as analyzed using linear multiple regression, equal to Δ Z-score B-vitamin - Δ Z-score placebo

Crude model : adjusted for baseline cognitive Z-score; Adjusted model: adjusted for baseline cognitive Z-score, age, sex, level of education, ApoE4 status, baseline homocysteine concentration, baseline body mass index, physical activity, smoking status.

|  |  | Treatment effect^1^ | | | Overall interaction^2^ | Tertiles pairwise comparison^3^ | | |
| --- | --- | --- | --- | --- | --- | --- | --- | --- |
|  |  | Crude | Adjusted | p-value | p-value | low vs middle | low vs high | middle vs high |
| Episodic memory | |  |  |  | 0.97 |  |  |  |
|  | Low tertile | 0.22±0.15 | 0.13±0.15 | 0.96 |  | diff=0.02±0.21 p=0.91 | diff=0.05±0.21 p=0.80 | diff=0.03±0.21 p=0.88 |
|  | Middle tertile | 0.11±0.14 | 0.10±0.15 | 0.98 |  |  |  |  |
|  | High tertile | 0.06±0.15 | 0.07±0.15 | 1.00 |  |  |  |  |
| Attention & working memory | |  |  |  | 0.76 |  |  |  |
|  | Low tertile | 0.07±0.15 | 0.10±0.16 | 0.99 |  | diff=0.09±0.23 p=0.68 | diff=0.17±0.24 p=0.46 | diff=0.07±0.22 p=0.74 |
|  | Middle tertile | 0.01±0.15 | 0.01±0.16 | 1.00 |  |  |  |  |
|  | High tertile | -0.03±0.15 | -0.07±0.16 | 1.00 |  |  |  |  |
| Information processing speed | |  |  |  | 0.57 |  |  |  |
|  | Low tertile | -0.01±0.12 | -0.03±0.12 | 1.00 |  | diff=0.15±0.17 p=0.37 | diff=0.17±0.17 p=0.34 | diff=0.01±0.17 p=0.94 |
|  | Middle tertile | 0.22±0.12 | 0.12±0.12 | 0.90 |  |  |  |  |
|  | High tertile | 0.25±0.12 | 0.14±0.12 | 0.87 |  |  |  |  |
| Executive functioning | |  |  |  | 0.65 |  |  |  |
|  | Low tertile | 0.14±0.13 | 0.04±0.13 | 1.00 |  | diff=0.03±0.18 p=0.88 | diff=0.16±0.18 p=0.39 | diff=0.13±0.18 p=0.46 |
|  | Middle tertile | 0.05±0.13 | 0.07±0.13 | 0.99 |  |  |  |  |
|  | High tertile | 0.26±0.13 | 0.20±0.13 | 0.62 |  |  |  |  |

^2^ The overall interaction indicates similarity of treatment effects in the low, middle and high omega-3 fatty acid tertiles.

^3^ The pairwise comparison tests for differences in treatment effects between omega-3 fatty acid tertiles.

**Supplementary table 4:** Changes in domain-specific cognitive Z-scores following B-vitamin versus placebo supplementation according to DHA status tertile.

Data available for n=191 participants. Data is presented as mean β±SEM. Abbreviations: DHA: docosahexaenoic acid

^1^ Treatment effect is the difference in change in Z-score over time between the B-vitamin and placebo treatment groups within an omega-3 fatty acid tertile as analyzed using linear multiple regression, equal to Δ Z-score B-vitamin - Δ Z-score placebo.

Crude model : adjusted for baseline cognitive Z-score; Adjusted model: adjusted for baseline cognitive Z-score, age, sex, level of education, ApoE4 status, baseline homocysteine concentration, baseline body mass index, baseline smoking status, baseline physical activity.

^2^ The overall interaction indicates similarity of treatment effects in the low, middle and high omega-3 fatty acid tertiles.

^3^ The pairwise comparison tests for differences in treatment effects between omega-3 fatty acid tertiles.

|  |  | Treatment effect^1^ | | | Overall interaction^2^ | Tertiles pairwise comparison^3^ | | |
| --- | --- | --- | --- | --- | --- | --- | --- | --- |
|  |  | Crude | Adjusted | p-value | p-value | low vs middle | low vs high | middle vs high |
| Episodic memory | |  |  |  | 0.40 |  |  |  |
|  | Low tertile | 0.19±0.15 | 0.11±0.15 | 0.98 |  | diff=0.11±0.21 p=0.61 | diff=0.19±0.22 p=0.39 | diff=0.30±0.22 p=0.18 |
|  | Middle tertile | 0.06±0.14 | 0.00±0.15 | 1.00 |  |  |  |  |
|  | High tertile | 0.24±0.16 | 0.30±0.16 | 0.42 |  |  |  |  |
| Attention & working memory | |  |  |  | 0.95 |  |  |  |
|  | Low tertile | -0.01±0.16 | 0.02±0.16 | 1.00 |  | diff=0.06±0.22 p=0.78 | diff=0.00±0.24 p=0.99 | diff=0.07±0.23 p=0.78 |
|  | Middle tertile | -0.06±0.15 | -0.05±0.16 | 1.00 |  |  |  |  |
|  | High tertile | 0.03±0.16 | 0.02±0.17 | 1.00 |  |  |  |  |
| Information processing speed | |  |  |  | 0.24 |  |  |  |
|  | Low tertile | 0.13±0.13 | 0.10±0.12 | 0.97 |  | diff=0.19±0.17 p=0.27 | diff=0.10±0.18 p=0.57 | diff=0.30±0.18 p=0.10 |
|  | Middle tertile | 0.00±0.12 | -0.09±0.12 | 0.97 |  |  |  |  |
|  | High tertile | 0.29±0.13 | 0.20±0.13 | 0.62 |  |  |  |  |
| Executive functioning | |  |  |  | 0.50 |  |  |  |
|  | Low tertile | 0.14±0.13 | 0.06±0.13 | 1.00 |  | diff=0.02±0.18 p=0.92 | diff=0.21±0.19 p=0.28 | diff=0.19±0.19 p=0.32 |
|  | Middle tertile | 0.08±0.13 | 0.07±0.13 | 0.99 |  |  |  |  |
|  | High tertile | 0.31±0.14 | 0.26±0.14 | 0.40 |  |  |  |  |
